# A preliminary evaluation of a fast, low-cost, and high-throughput nucleic acid extraction method for bacterial microbiota profiling in low-microbial biomass samples

**DOI:** 10.1038/s41598-024-84682-9

**Published:** 2025-01-15

**Authors:** Xiangning Bai, Simon Rayner, Linda Ellevog Skuggen, Kine Kragl Engseth, Pål Marius Bjørnstad, Marianne Dalland, Gregor D. Gilfillan, Magnar Bjørås, Andreas Matussek

**Affiliations:** 1 Department of Microbiology, Division of Laboratory Medicine, Oslo University Hospital, 0372 Oslo, Norway; 2 Department of Microbiology, Division of Laboratory Medicine, Institute of Clinical Medicine, University of Oslo, 0372 Oslo, Norway; 3 Division of Infectious Diseases, Department of Medicine Huddinge, Karolinska Institutet, 14186 Stockholm, Sweden; 4 Department of Medical Genetics, Oslo University Hospital and University of Oslo, 0450 Oslo, Norway; 5 Faculty of Chemistry, Biotechnology and Food Science, Norwegian University of Life Sciences (NMBU), 1433 Ås, Norway; 6 Department of Clinical and Molecular Medicine (IKOM), Norwegian University of Science and Technology (NTNU), 7491 Trondheim, Norway; 7 Centre for Embryology and Healthy Development, University of Oslo, 0373 Oslo, Norway; 8 Division of Clinical Microbiology, Department of Laboratory Medicine, Karolinska Institutet, 141 52 Stockholm, Sweden

**Keywords:** Microbiota, Bacteria, 16S rRNA gene sequencing, Low-microbial biomass, Respiratory samples, Nucleic acid extraction, Metagenomics, Microbial communities

## Abstract

The respiratory tract is colonized with low-density microbial communities, which have been shown to impact human respiratory health through microbiota–host interactions. However, a lack of fast and cost-effective nucleic acid extraction method for low-microbial biomass samples hinders investigation of respiratory microbiota. Here, we performed a pilot study to assess the suitability of the NAxtra nucleic acid extraction protocol for profiling bacterial microbiota in respiratory samples. A small number of nasopharyngeal aspirate (*n* = 8), nasal swab (*n* = 8), and saliva samples (*n* = 8) were collected, nucleic acids were isolated using the NAxtra protocol, and 16 S rRNA gene sequencing was performed to characterize bacterial microbiota, which were compared to the same sample types from previous studies using other protocols. The bacterial composition in nasal and saliva samples were consistent with previous reports. Saliva microbiota was significantly richer than nasal microbiota and varied less among individual samples than nasal microbiota. Bacterial composition in nasal samples was distinct from nasopharyngeal aspirates, but closer to saliva samples. A sequencing depth of 50,000 reads/sample was sufficient for microbiota profiling in low biomass respiratory samples. Our pilot study indicates the potential of the NAxtra protocol for bacterial microbiota characterization of low-microbial biomass samples and supports a more comprehensive study to fully evaluate the value of the NAxtra protocol in microbiota research and clinical diagnostics of respiratory pathogens.

## Introduction

A microbiota refers to a collection of microorganisms within a specific environment and encompasses bacteria, viruses, archaea, and fungi. The corresponding microbiome refers to this collection of microorganisms and their genomes^1^. The microbiota can be classified within localized regions, such as the gut and respiratory tract, and their composition is found to vary widely across different sites^1^. As bacteria are the most abundant component of the microbiota^2^, most studies are focused on bacterial microbiota at body sites that have high microbial biomass, such as the gut. The gut microbiota has thus been widely studied and shown to play important roles in maintaining human health^3^. More recently, there has been growing interest in the role of microbiota at low-microbial biomass body sites, such as the respiratory tract^4^. The microbial communities within the respiratory tract contain potential respiratory pathogens, but also act as a first-line defense against respiratory infections such as COVID-19, possibly modulating the outcomes of respiratory disease^5^. A growing number of studies^6^, including our own^7^, shows that the respiratory microbiota presents a significant therapeutic and prophylactic potential against respiratory infections and severe patient outcome. However, one of the challenges in analyzing low-microbial biomass respiratory microbiota samples is the lack of reliable, sensitive, fast, and cost-effective nucleic acid isolation techniques that can be used for large-scale research and clinical settings to advance our understanding of respiratory microbiota in relation to human health.

Numerous methods and commercial kits have been developed for nucleic acid extraction, for example the QIAamp DNA Microbiome kit, the Molzym Ultra-Deep Microbiome Prep kit, and the MasterPure Complete DNA and RNA Purification kit, which can be applied to various sample types including low-microbial biomass swabs and fluids. However, these commercial kits are often costly, and some use filter-based spin-column methods which can be difficult or impossible to automate using liquid handling robots to achieve higher throughput. Cost-effective, high-throughput alternatives exhibiting comparable or improved performance to the currently available commercial kits would make larger studies feasible and accelerate advancements in microbiota research. In Norway, during the COVID-19 pandemic, a test kit was initially developed for rapid isolation of viral RNA to detect SARS-CoV-2 infection with a sensitivity comparable to available commercial kits^8^. The extraction procedure, based on the use of NAxtra magnetic nanoparticles is low-cost, robust, and reliable, providing high-quality nucleic acids within a short time. When automated, nucleic acid extraction can be completed within 14 min for up to 96 samples on the robot systems KingFisher Flex and KingFisher Duo Prime^9^. It can also be done on higher-throughput liquid handling robots such as the Tecan Fluent® Automated Workstation, which could process 288 samples in one run within an hour. The NAxtra protocol was subsequently adapted and further validated for nucleic acid extraction from other types of viruses^8^, as well as mammalian DNA and RNA^9^. However, the suitability of this protocol for other biological applications, including bacterial microbiota characterization, remains to be assessed.

Here we report the outcome of a simple pilot study to establish whether further adaptation of the NAxtra protocol is possible for the broader application of profiling bacterial microbiota in low-microbial biomass samples. Here, rather than performing an expensive and time-consuming comprehensive study, we carried out a pilot study of a small number of (i) low biomass nasopharyngeal aspirates and nasal swabs, and (ii) saliva samples, which contain relatively higher microbial biomass. We evaluated these samples by characterizing their bacterial microbiota using 16 S rRNA gene sequencing, which we then compared to the same sample types from previous studies using other extraction protocols. In addition, we compared the bacterial microbiota profiles from different respiratory sample types in this study.

## Materials and methods

### Ethics

Samples used in this study were collected in the context of routine diagnostics, and the assays were performed on residual anonymized patient materials from a Norwegian-accredited diagnostic service laboratory supporting diagnostic processes, including nucleic acid extractions for standard RT-PCR assays, thereby not requiring informed consent and ethics committee approval, in compliance with IRB regulations and Norwegian National Legislation of Infection Prevention Act [Smittevernloven] (§ 3–7): Laboratories and institutions may carry out methodological testing using available sample material without the consent of those who have given the samples if the purpose of the testing is to develop new methods or improve existing methods for the identification and description of a communicable disease. https://lovdata.no/dokument/NL/lov/1994-08-05-55?q=Smittevernloven.

### Sample collection and nucleic acid extraction

Nasopharyngeal aspirate and nasal swab samples (*n* = 8 each), and saliva samples (*n* = 8) were randomly selected from routine diagnostic sampling at Oslo University Hospital (OUH), Oslo, Norway, and treated anonymously. Total nucleic acid was extracted using the NAxtra™ nucleic acid extraction kit (Lybe Scientific) according to manufacturer’s instruction^8^ https://lybescientific.com/products/ on a Tecan Fluent® Automated Workstation at the laboratory in the Department of Microbiology at OUH, with minor modification, i.e., the elute buffer (water) was decreased from 100 µl in the original protocol to 80 µl to increase the DNA concentrations. The sample input volume was 100 µl. The DNA concentrations in the elutes were measured using a Qubit™ dsDNA HS kit on a Qubit 3.0 Fluorometer (Life Technologies, Darmstadt, Germany), and 2 µl DNA eluate was used as input volume.

### 16 S rRNA gene sequencing

The V3-V4 region of the 16 S rRNA gene was amplified based on the two-step PCR procedure (25 cycles were used for first PCR and 8 cycles for second PCR), as described in the Illumina application note^10^ using primer sequences derived from Klindworth et al.^11^. Positive control ZymoBIOMICS Microbial Community DNA Standard II (Log distribution) (Zymo Research, Irvine, CA, USA) was used to control for PCR amplification of 16 S rRNA gene and sequencing, and negative control (water) was used to identify and remove potential contaminations (i.e., taxa present in DNA elution buffer water). The final libraries were verified using the Tapestation 4200 with D1000 reagents (Agilent, Santa Clara, USA) and quantified on a SpectraMax M3 fluorometric plate reader (Molecular Devices, San Jose, CA, USA) using Quant-it HS Assay reagents (Thermo Fisher Scientific, Waltham, MA, USA). Libraries were pooled based on their concentrations and then sequenced on an Illumina MiSeq platform (Illumina, San Diego, CA, USA) with 300 bp paired end reads (v3 reagents). 20% PhiX control library was added to the libraries, and cluster density was reduced to 80% of regular levels. Base calling and production of demultiplexed FASTQ files were performed by running RTA v1.18.54.4 and bcl2fastq v2.20.0.422.

### Bioinformatics and statistics

The demultiplexed FASTQ files were analyzed using **QIIME2 version 2022.8** implemented in a SLURM script. Reads were demultiplexed and adapters were trimmed using **cutadapt** with forward and reverse sequences CCTACGGGNGGCWGCAG and GACTACHVGGGTATCTAATCC respectively. To remove low-quality calls at the ends of the reads, adapter-trimmed reads were truncated at 250 bp for the forward read and 190 bp for the reverse read. Denoising and amplicon sequence variant (ASV) inference was performed using **DADA2**. Taxonomic classification of the ASVs was then carried out with the *feature-classifier classify-sklearn* command with SILVA release 138 as the reference. A phylogenetic tree was estimated using the *align-to-tree-mafft-fasttree* command, which generates a **MAFFT** alignment from reads, and **fasttree** was used to estimate a phylogenetic tree from these alignments. To remove possible contaminations (taxa present in the negative control water sample), a vector was created with all ASVs except the ones present in the negative control. This vector was then applied to the ‘*prune_taxa()*’ function from the R **phyloseq** package on the ‘*phyloseq*’ object. Alpha diversity indices, including observed ASVs and Shannon index, were calculated using the **phyloseq** package with the function ‘*estimate_richness ()*’. Differences in alpha diversity indexes between groups were tested with the Kruskal-Wallis test, followed by a Wilcoxon rank sum test when statistically significant indexes were identified by Kruskal-Wallis. Benjamini–Hochberg correction was used to adjust p-values from the Wilcoxon rank sum test for multiple testing. Beta diversity analysis was based on Bray–Curtis distances determined using the phyloseq package. Samples were clustered according to bacterial composition using principal coordinates analysis (PCoA) based on the Bray–Curtis distances. Permutational multivariate analysis of variance (PERMANOVA) was performed to test the differences in bacterial composition between groups using the **vegan** package (with the *Adonis* function) with the Bray–Curtis dissimilarity method. The QIIME2 analysis SLURM script and downstream R analysis and visualization scripts are available on GitHub at (https://github.com/CBGOUS/baisi24).

## Results

### DNA recovery from respiratory samples using the NAxtra protocol

Saliva samples showed higher DNA yield ranging from 0.242 to 17.8 ng/µl among individual samples, as compared to nasopharyngeal aspirates ranging from 0.286 to 12.8 ng/µl, and nasal swabs ranging from 0.058 to 4.44 ng/µl (Table 1).

**Table 1:** DNA yield of respiratory samples using the NAxtra protocol.

Sample type	Sample ID	DNA concentration (ng/µl)
Saliva	S1	7.35
	S2	0.242
	S3	5.27
	S4	2.82
	S5	6.12
	S6	17.8
	S7	3.55
	S8	1.11
Nasal swab	1B	4.44
	2B	0.525
	3B	0.058
	4B	0.456
	5B	0.31
	6B	0.152
	7B	0.279
	8B	0.148
Nasopharyngeal aspirate	1A	0.286
	2A	0.91
	3A	NA
	4A	1.26
	5A	12.8
	6A	1.17
	7A	1.46
	8A	1.17

NA: DNA concentration out of detection range on Qubit (below 0.005 ng/µl).

### Microbiota diversity and composition in respiratory samples

**Fig. 1:**
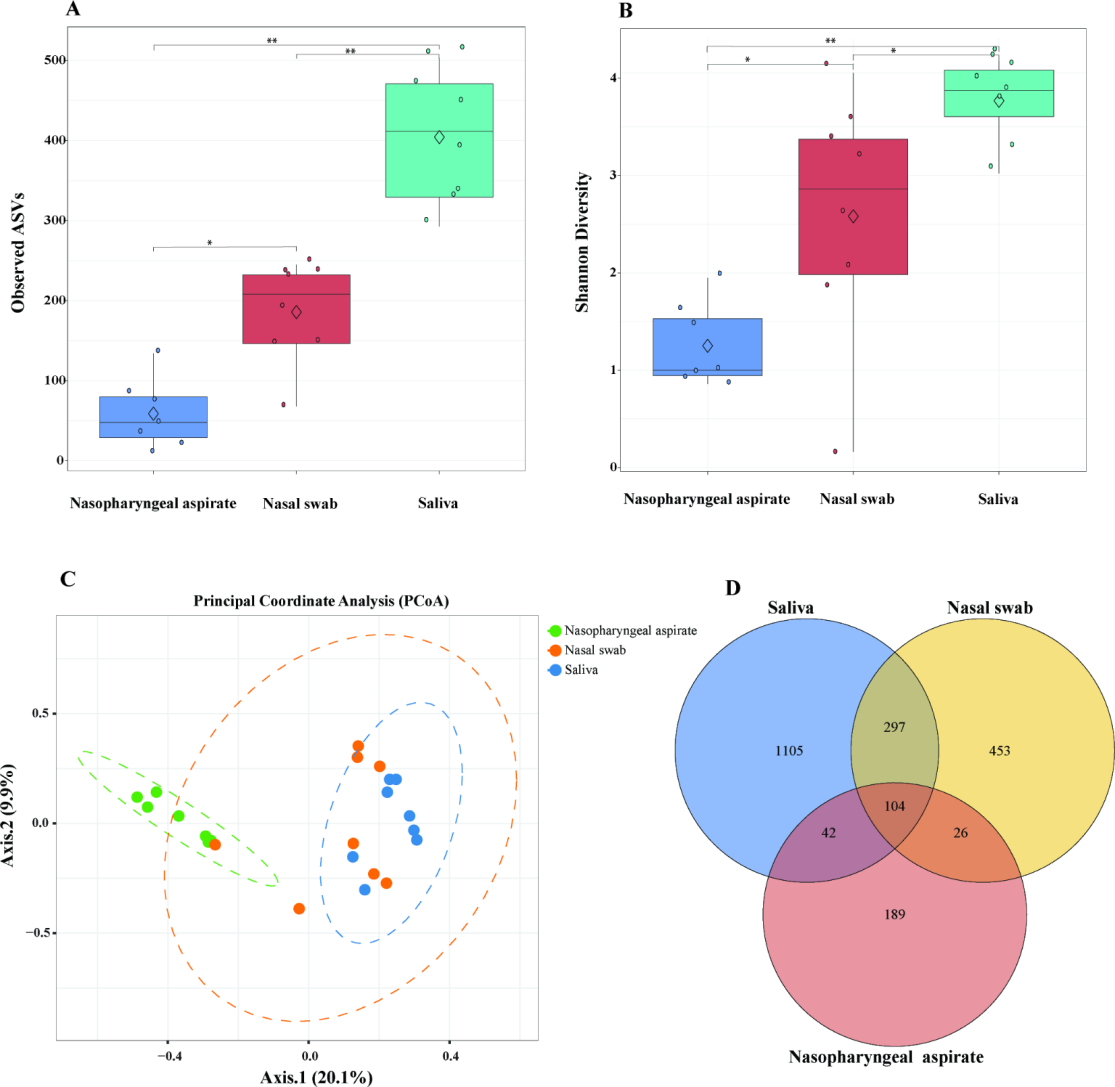
Bacterial microbiota diversity and composition in different samples. Box plots showing the alpha diversity indices represented by (**A**) observed amplicon sequence variants (ASVs), and (**B**) Shannon diversity. Boxes represent the interquartile range between the 25th and 75th percentiles, and the horizontal line inside the box denotes the median value, and the diamond represents the mean value. Asterisks refer to the Benjamini–Hochberg corrected p value, where *p < 0.05, **p < 0.001. (**C**) Beta diversity illustrated by principal coordinates analysis (PCoA) based on Bray–Curtis distance. (**D**) Venn diagram showing the numbers of common and distinct ASVs among different sample types.

One nasopharyngeal aspirate sample 2A failed in 16 S rRNA gene amplification and thus was excluded in the subsequent sequencing and analysis. After quality filtering, including removal of possible contaminations (i.e., taxa present in the negative control water), an average of 334,315 reads per sample was produced across 23 samples. Saliva samples showed a statistically significant higher bacterial alpha diversity measured by observed ASVs richness and Shannon diversity index, compared to the nasopharyngeal aspirates and nasal swabs (B-H adjusted *p* < 0.05, Wilcoxon rank sum test) (Fig. 1A and B). A PCoA plot showed that nasopharyngeal aspirates were separated from nasal swabs and saliva samples, while nasal swabs and saliva samples clustered more closely (*p* < 0.05, PERMANOVA) (Fig. 1C). A total of 104 ASVs were common to all three sample types, while saliva and nasal swabs shared additional 297 ASVs, saliva and nasopharyngeal aspirates shared additional 42 ASVs, nasal swabs and nasopharyngeal aspirates shared additional 26 ASVs (Fig. 1D).

**Fig. 2:**
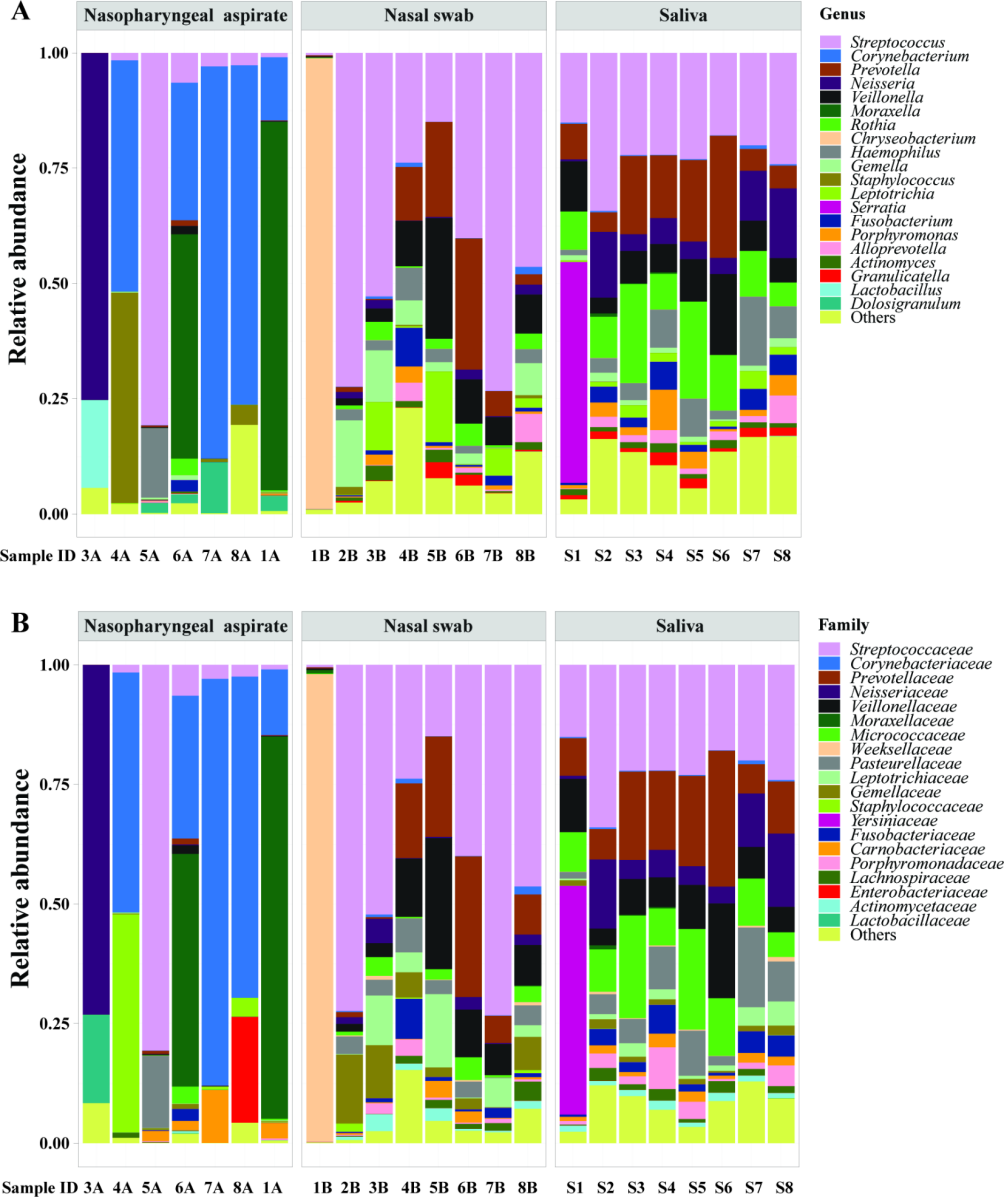
Abundance of bacterial taxa in different samples. (**A**) Genus level. (**B**) Family level. The 20 most abundant genera and families are indicated in different colors, the remaining taxa are binned into the ‘Others’ category.

At the genus level, the most abundant taxon in saliva samples was *Streptococcus*, followed by *Rothia*, *Prevotella*, *Veillonella*,* Neisseria*, *and Haemophilus* (Fig. 2A). In nasal swabs, *Streptococcus* was the most abundant, followed by *Chryseobacterium*, *Prevotella*, *Veillonella*, *Gemella* and *Leptotrichia*. The most abundant taxon in nasopharyngeal aspirates was *Corynebacterium*, followed by *Moraxella*, *Streptococcus*, *Staphylococcus*, and *Dolosigranulum.* We observed higher similarity of bacterial composition among individual saliva samples than individual nasopharyngeal aspirate and nasal swab (Fig. 2A). A similar tendency was observed at the family level (Fig. 2B). We observed high abundance of certain gram-positive bacteria in different respiratory samples, for example, *Streptococcus*, *Rothia*, *Corynebacterium* and *Staphylococcus*, in line with previous findings in such samples^12^. These data suggest that the NAxtra protocol works efficiently on bacterial microbiota profiling, without additional lysozyme treatment specifically for gram-positive bacteria.

### Impact of sequencing depth on nasal and saliva microbiota characterization

**Fig. 3:**
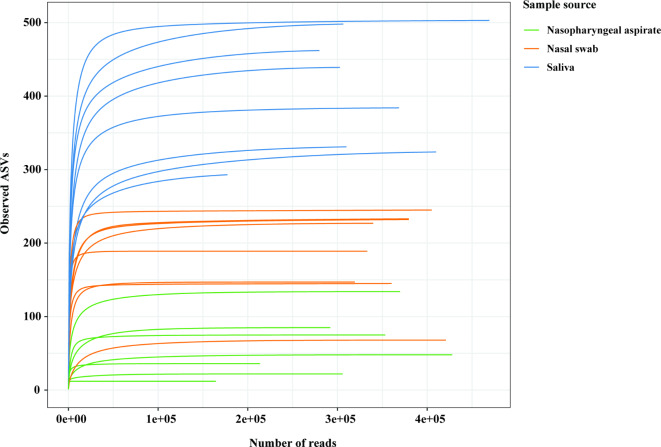
Rarefaction curves. The curve is created for each sample to assess the saturation at different sequencing depths for recovery of bacterial taxa presented by amplicon sequence variants (ASVs). Nasopharyngeal aspirate and nasal swab samples reach a plateau more rapidly (~ 10,000 reads) compared to saliva samples (~ 50,000 reads).

Rarefaction curves are used as a qualitative method to estimate the bacteria richness as a function of sequencing depth. Our results showed that the rarefaction curves reached a plateau at a higher sequencing depth for saliva samples than for nasopharyngeal aspirates and nasal swabs (Fig. 3). For nasopharyngeal aspirates and nasal swabs, the rarefaction curves reached a plateau at a sequencing depth of 10,000 reads for most samples. While for saliva samples, approximately 50,000 reads per sample were required to capture the full set of ASVs.

## Discussion

In this study, we performed a preliminary assessment of the suitability of a fast, low-cost, robust and high-throughput NAxtra™ nucleic acid extraction kit^8^ for characterizing bacterial microbiota in low-microbial biomass respiratory samples using 16 S rRNA gene sequencing. DNA concentrations and total yields of nasal samples in this study were higher than those reported by Giske et al., where the same input volume of the original sample (100 µl) and a lower volume of elution buffer (50 µl) was used^13^. DNA yields isolated from saliva samples using the NAxtra protocol were comparable to those reported using other methods with the same saliva sample input volume (100 µl)^13^. Saliva samples showed higher DNA density than nasal samples, in line with the report from Giske et al.^13^. DNA yields within the same sample types were variable in this study, as reported elsewhere for nasal samples^14^. It should be noted that other factors may also impact the DNA yield, including variations in sampling populations, sampling methods, and storage buffers^15^. Thus, results from different studies should be compared with caution, and further comparisons between NAxtra and other methods are required to validate the results. Importantly, microbiota profiling showed a high abundance of certain gram-positive bacteria e.g., *Streptococcus*,* Staphylococcus* in our tested samples, suggesting that the NAxtra protocol and NAxtra lysis buffer works efficiently on gram-positive bacteria without additional use of lysozyme.

We compared the diversity and composition of the nasal, nasopharyngeal and saliva microbiota. The results showed that the bacterial microbiota of saliva samples was significantly richer than that of nasal swabs and nasopharyngeal aspirates, and saliva bacterial microbiota varied less among individual samples than did nasal and nasopharyngeal microbiota. This aligns with findings from studies comparing the oral and nasal bacterial microbiota from same participants^16,17^, although the saliva and nasal samples were from different participants in this study. It has been shown that distinct microbiota profiles exist at different sites along the respiratory tract, for instance, bacteria *Prevotella*, *Streptococcus*, *Veillonella*, *Rothia* are predominant in the oropharynx, while in the nasopharynx, the dominant bacteria are *Moraxella*, *Corynebacterium* and *Neisseria*^12^. Similarly, our study showed the most abundant bacteria in saliva samples were *Streptococcus*,* Rothia*, *Prevotella* and *Veillonella*; while in nasopharyngeal aspirates, *Corynebacterium* and *Moraxella* were most abundant. Interestingly, we observed that bacterial composition in nasal swabs was distinct from nasopharyngeal aspirates, while closer to saliva. This could be due to that the anterior nasal cavity is physiologically closer to oral cavity than nasopharynx, the niche-specific selective growth conditions may shape the microbial communities along the respiratory tract^4,18^. Our finding was supported by another study reporting a related but distinct bacterial microbiota composition between the anterior nares and nasopharynx^19^, yet few existing studies have so far simultaneously compared microbiota among nasal cavity, nasopharynx, and oral cavity. As the metadata was unavailable for the study participants, we could not make any further biological interpretation on the microbiota differences observed amongst different sample types.

Few studies have assessed the 16 S rRNA gene sequencing depth for characterization of low-microbial biomass samples. In this study, given the small number of samples, we obtained ultra-deep sequencing dataset with an average coverage of 334,315 clean reads per sample, allowing evaluation of the required sequencing depth for low-microbial biomass samples. Our results showed that saliva samples reached a plateau at a higher sequencing depth than nasal samples, which is reasonable as samples with higher microbial density and diversity would require greater sequencing depth. In general, our study showed that a 16 S rRNA gene sequencing depth of 50,000 reads per sample is sufficient to capture a full bacteria composition in nasal and saliva samples. This allows the high throughput characterization of large numbers of samples in a single 16 S rRNA gene sequencing run. Conversely, this suggests that the cost of DNA extraction could constitute a significant portion of the total expenses in studies with large sample sizes. Therefore, our extraction protocol, with its low cost, could make larger studies more feasible.

We acknowledge limitations of this pilot study. Firstly, the sample size was small and there are no technical replicates, thus the comparisons of bacterial community from different sample types lack statistical support. Secondly, we did not compare the NAxtra protocol with other DNA extraction methods in this study, thus we were unable to draw comparisons between extraction methods for bacterial microbiota profiling in this study, although its sensitivity and selectivity has been proven to be comparable to products on the market in detecting viral and mammalian nucleic acids^8,9^. Thirdly, due to the restricted metadata associated with the samples, the biological interpretation of this study is limited. Fourthly, since we were using low-microbial biomass samples which produced accordingly low DNA yields, we were unable to reliably measure the purity of extracted DNA and ascertain any effect this may have had on DNA amplification. Nevertheless, from the outset, our goal was not to perform a comprehensive study to fully evaluate the protocol, for example, in a high throughput clinical setting, but rather to establish whether such a comprehensive study was justified. As such, our results indicate that a larger scale study containing replicates and comparing different extraction protocols is indeed warranted and could be carried out by a larger group with access to more resources. This would further verify our preliminary findings that indicate the potential of NAxtra protocol for large-scale microbiota studies and clinical diagnostics of pathogens in respiratory and other low biomass samples.

In conclusion, we report an assessment of a new nucleic acid extraction method on bacterial microbiota profiling in low-microbial biomass respiratory samples. The bacterial composition identified using the NAxtra protocol is comparable to that in similar sample types reported elsewhere using different extraction protocols. Given the simplicity, reduced time, low-cost, and high throughput, the NAxtra method has the potential to serve as an attractive option for large-scale microbiota research and routine diagnostics of respiratory pathogens in clinical settings. However, a more comprehensive study is required to clearly establish its benefits for microbiota analysis as compared to other protocols.

## Data Availability

The raw sequencing data have been submitted to the NCBI Sequence Read Archive (SRA) under the BioProject ID PRJNA1080104.

## References

[1] Hou, K. et al. Microbiota in health and diseases. *Signal. Transduct. Target. Ther.* **7**, 135. 10.1038/s41392-022-00974-4 (2022). 35461318 10.1038/s41392-022-00974-4 PMC9034083

[2] Matijasic, M. et al. Gut microbiota beyond Bacteria-Mycobiome, Virome, Archaeome, and eukaryotic parasites in IBD. *Int. J. Mol. Sci.* **21**10.3390/ijms21082668 (2020). 10.3390/ijms21082668 PMC7215374 32290414

[3] Hillman, E. T., Lu, H., Yao, T. & Nakatsu, C. H. Microbial ecology along the gastrointestinal tract. *Microbes Environ.* **32**, 300–313. 10.1264/jsme2.ME17017 (2017). 29129876 10.1264/jsme2.ME17017 PMC5745014

[4] Man, W. H., de Steenhuijsen Piters, W. A. & Bogaert, D. The microbiota of the respiratory tract: Gatekeeper to respiratory health. *Nat. Rev. Microbiol.* **15**, 259–270. 10.1038/nrmicro.2017.14 (2017). 28316330 10.1038/nrmicro.2017.14 PMC7097736

[5] Merenstein, C., Bushman, F. D. & Collman, R. G. Alterations in the respiratory tract microbiome in COVID-19: Current observations and potential significance. *Microbiome* **10**, 165. 10.1186/s40168-022-01342-8 (2022). 36195943 10.1186/s40168-022-01342-8 PMC9532226

[6] Di Simone, S. K., Rudloff, I., Nold-Petry, C. A., Forster, S. C. & Nold, M. F. Understanding respiratory microbiome-immune system interactions in health and disease. *Sci. Transl. Med.* **15**, eabq5126. 10.1126/scitranslmed.abq5126 (2023). 36630485 10.1126/scitranslmed.abq5126

[7] Bai, X. et al. Characterization of the Upper Respiratory bacterial microbiome in critically ill COVID-19 patients. *Biomedicines* **10**. 10.3390/biomedicines10050982 (2022). 10.3390/biomedicines10050982 PMC9138573 35625719

[8] Ravlo, E. et al. A fast, low-cost, robust and high-throughput method for viral nucleic acid isolation based on NAxtra magnetic nanoparticles. *Sci. Rep.* **13**, 11714. 10.1038/s41598-023-38743-0 (2023). 37474666 10.1038/s41598-023-38743-0 PMC10359305

[9] Starheim, E. J. et al. NAxtra magnetic nanoparticles for low-cost, efficient isolation of mammalian DNA and RNA. *Sci. Rep.* **13**, 20836. 10.1038/s41598-023-46868-5 (2023). 38012172 10.1038/s41598-023-46868-5 PMC10682382

[10] 16SMetagenomicSequencingLibrary Preparation. https://www.illumina.com/content/dam/illumina-support/documents/documentation/chemistry_documentation/16s/16s-metagenomic-library-prep-guide-15044223-b.pdf

[11] Klindworth, A. et al. Evaluation of general 16S ribosomal RNA gene PCR primers for classical and next-generation sequencing-based diversity studies. *Nucleic Acids Res.* **41**, e1. 10.1093/nar/gks808 (2013). 22933715 10.1093/nar/gks808 PMC3592464

[12] Natalini, J. G., Singh, S. & Segal, L. N. The dynamic lung microbiome in health and disease. *Nat. Rev. Microbiol.* **21**, 222–235. 10.1038/s41579-022-00821-x (2023). 36385637 10.1038/s41579-022-00821-x PMC9668228

[13] Biesbroek, G. et al. Deep sequencing analyses of low density microbial communities: Working at the boundary of accurate microbiota detection. *PLoS One*. **7**, e32942. 10.1371/journal.pone.0032942 (2012). 22412957 10.1371/journal.pone.0032942 PMC3295791

[14] Silva, R. C. D. et al. Comparison of DNA extraction methods for COVID-19 host genetics studies. *PLoS One*. **18**, e0287551. 10.1371/journal.pone.0287551 (2023). 37903126 10.1371/journal.pone.0287551 PMC10615309

[15] Claassen-Weitz, S. et al. Optimizing 16S rRNA gene profile analysis from low biomass nasopharyngeal and induced sputum specimens. *BMC Microbiol.* **20**10.1186/s12866-020-01795-7 (2020). 10.1186/s12866-020-01795-7 PMC7218582 32397992

[16] Lemon, K. et al. Comparative analyses of the bacterial microbiota of the human nostril and oropharynx. *mBio* **1**10.1128/mBio.00129-10 (2010). 10.1128/mBio.00129-10 PMC2925076 20802827

[17] Bassis, C. M. et al. Analysis of the upper respiratory tract microbiotas as the source of the lung and gastric microbiotas in healthy individuals. *Mbio* **6**. 10.1128/mBio.00037-15 (2015). 10.1128/mBio.00037-15 PMC4358017 25736890

[18] Flynn, M. F., Kelly, M. & Dooley, J. S. G. Nasopharyngeal swabs vs. nasal aspirates for respiratory virus detection: A systematic review. *Pathogens* **10**. 10.3390/pathogens10111515 (2021). 10.3390/pathogens10111515 PMC8620365 34832670

[19] Luna, P. N. et al. The association between anterior nares and nasopharyngeal microbiota in infants hospitalized for bronchiolitis. *Microbiome* **6**. 10.1186/s40168-017-0385-0 (2018). 10.1186/s40168-017-0385-0 PMC5751828 29298732

